# *freesurfer*: Connecting the Freesurfer software with R

**DOI:** 10.12688/f1000research.14361.1

**Published:** 2018-05-16

**Authors:** John Muschelli, Elizabeth Sweeney, Ciprian M. Crainiceanu

**Affiliations:** 1 Department of Biostatistics, Johns Hopkins Bloomberg School of Public Health, Baltimore, MD, 21205, USA; 2 Flatiron Health, New York, NY, 10010, USA

**Keywords:** freesurfer, r, neuroconductor, neuroimaging

## Abstract

We present the package *freesurfer*, a set of R functions that interface with Freesurfer, a commonly-used open-source software package for processing and analyzing structural neuroimaging data, specifically T1-weighted images. The *freesurfer* package performs operations on nifti image objects in R using command-line functions from Freesurfer, and returns R objects back to the user. *freesurfer *allows users to process neuroanatomical images and provides functionality to convert and read the output of the Freesurfer pipelines more easily, including brain images, brain surfaces, and Freesurfer output tables.

## Introduction

Freesurfer is a commonly-used software for processing and analyzing anatomical neuroimaging data ^1^, developed by the Laboratory for Computational Neuroimaging at the Athinoula A. Martinos Center for Biomedical Imaging. This software provides open-source, command-line tools for image processing tasks such as brain extraction/skull-stripping ^2^, bias-field correction ^3^, segmentation of structures within the brain ^4, 5^, and image registration ^6, 7^. In addition to these functions, Freesurfer has functions that perform fully-automated pipelines for the user.

There exist a number of R packages for reading and manipulating image data, including **AnalyzeFMRI** ^8^ and **fmri** ^9^, which analyze functional magnetic resonance images (MRI) and perform spatial smoothing, **RNiftyReg** ^10^, which performs image registration, and **dpmixsim** ^11^ and **mritc** ^12^, which perform image clustering and segmentation (see the Medical Imaging CRAN task view for more information). These packages provide powerful tools for performing image analysis, but the neuroimaging community has additional tools that may perform better on a specific data set or provide more information than these R packages. Freesurfer provides methods that are not currently implemented in R, including surface-based registration and completely automated image segmentation pipelines. The **ANTsR** package is a currently unpublished R package where additional image analysis functionality has been implemented, but does not include all the functionality Freesurfer has. Moreover, having multiple options for image processing through R enables users to compare methods and provides the flexibility of using multiple packages to achieve a working data processing pipeline.

We provide an interface to users for the state-of-the-art anatomical processing implemented in Freesurfer, as well as a suite of tools that simplify analyzing the output of Freesurfer. The **freesurfer** package allows R users to perform a complete anatomical imaging analyses without necessarily learning Freesurfer-specific syntax, while keeping both the image processing and analysis within R.

## Methods

### R function setup

To use **freesurfer**, a working installation of Freesurfer is required (downloads available: http://freesurfer.net/fswiki/DownloadAndInstall). The following code was run using Freesurfer version “freesurfer-Darwin-lion-stable-pub-v5.3.0”. The Freesurfer version can be accessed using the **freesurfer** fs_version function. The path of Freesurfer must also be set. When using R from a shell environment, after the FREESURFER_HOME environment variable is set (which is done when installing Freesurfer), **freesurfer** will use this as the path to Freesurfer. If using R through a graphical user interface (GUI) such as RStudio (RStudio, Boston, MA), environmental variables and paths are not explicitly exported. Therefore, FREESURFER_HOME is not set and **freesurfer** will try the default directories of Mac OSX and Linux. Freesurfer is only available on Windows via a virtual machine. If the user did not perform a standard installation of Freesurfer, the path to Freesurfer can be specified using options(freesurfer.path="/path/to/freesurfer"). The have_fs function tests whether a user has a Freesurfer installation, returning a logical, which is useful for if statements within examples. If have_fs function returns is TRUE, the fs_dir function will return the directory of the Freesurfer installation.

### Operation

As per the https://surfer.nmr.mgh.harvard.edu/fswiki/DownloadAndInstall, Freesurfer only works on Linux or Mac operating systems. The work station should have at least a 2GHz processor, over 8GB of RAM, over 10GB hard drive space, and FSL ^13^ installed for certain functions.

### Structure of Freesurfer analyses

During the installation of Freesurfer, environment variables in addition to FREESURFER_HOME are set. One of these variables is SUBJECTS_DIR, which refers to a directory of the output of analysis from all subjects. The fs_subj_dir function will return the path to the Freesurfer subjects directory if it is set. This default setup of a subjects directory in Freesurfer allows users to simply specify a subject identifier to analyze, rather than a specific path or multiple intermediate files.

This setup may not be desirable if the user prefers to structure his or her data differently. For example, if data from multiple studies are present, these may be organized into different folders in different locations. Some functions in Freesurfer rely on the SUBJECTS_DIR variable to run. These functions take the subject name as the main argument rather than a file, which is more common. To provide flexibility to the user, **freesurfer** allows most functions to specify a file or different directory rather than specifying the subject.

One example is the asegstats2table Freesurfer function. Freesurfer performs segmentations of the anatomical image into different structures and has associated statistics for each region such as volume and mean intensity. The asegstats2table function transforms **a**natomical **seg**mentation **stat**istics from images into to a table. The default argument for asegstats2table is to pass in a subject name rather than a file. The **freesurfer** asegstats2table function allows the R user to specify the subject name, but also allows the user to alternatively specify a file name instead. This function will temporarily set SUBJECTS_DIR to a temporary directory, copy the file to that directory, execute the command, then reset the SUBJECTS_DIR variable. This provides a more flexible workflow, while not overriding the default directory set in SUBJECTS_DIR. This functionality allows users to have separate folders with subjects and read in the data by simply switching the subj_dir argument in the R function.

### Reconstruction pipeline in Freesurfer

The Freesurfer pipeline and analysis workflow for neuroanatomical images is designed to work with T1-weighted structural MRI of the brain. The full pipeline is implemented in the Freesurfer recon-all function, where the “recon” stands for reconstruction ( https://surfer.nmr.mgh.harvard.edu/fswiki/recon-all). The recon-all function is the main workhorse of Freesurfer and is the most commonly used. Using the -all flag in the the recon-all function performs over 30 different steps and takes 20–40 hours to fully process a subject ( https://surfer.nmr.mgh.harvard.edu/fswiki/recon-all). This process is the recommended way of fully processing a T1-weighted image in Freesurfer, and is implemented in the recon_all **freesurfer** function.

In the recon_all function, users must specify the input file (a T1-weighted image), the output directory (if different than SUBJECTS_DIR), and the subject identifier. The results will be written in the individual subject directory, a sub-directory of SUBJECTS_DIR. The syntax is:



recon_all(infile, outdir, subjid)
                    


If there are problems with the result of this processing, there are multiple steps where users can edit certain parts of the processing, such as brain extraction, where non-brain tissues are removed from the image. The remainder of the pipeline can be run after these steps are corrected. The full pipeline is broken down into 3 separate sets of steps, referred to as autorecon1, autorecon2, and autorecon3, which correspond to the same-named flags in recon-all used to initiate these steps. We have written wrapper functions autorecon1, autorecon2, and autorecon3, respectively, so users can run pieces of the pipeline if desired or restart a failed process after correction to the data.

### Imaging formats in **freesurfer** and R

The **freesurfer** package relies on the **oro.nifti** ^14^ package implementation of images (referred to as nifti objects) that are in the Neuroimaging Informatics Technology Initiative (NIfTI) format. For Freesurfer functions that require an image, the R **freesurfer** functions that call those Freesurfer functions will take in a file name or a nifti object. The R code will convert the nifti to the corresponding input required for Freesurfer. From the user’s perspective, the input/output process is all within R, with one object format ( nifti). The advantage of this approach is that the user can read in an image, do manipulations of the nifti object using standard syntax for arrays, and pass this object into the **freesurfer** R function. Thus, users can use R functionality to manipulate objects while seamlessly passing these object to Freesurfer through **freesurfer**.

Other Freesurfer functions require imaging formats other than NIfTI, such as the Medical Imaging NetCDF (MINC) format. The Freesurfer installation provides functions to convert from MINC to NIfTI formats and these are implemented in functions such as nii2mnc and mnc2nii in R. Moreover, the mri_convert Freesurfer function has been interfaced in **freesurfer** (same function name), which allows for a more general conversion tool of imaging types for R users than currently implemented in native R. Thus, many formats can be converted to NIfTI and then read into R using the readNIfTI function from **oro.nifti**.

## Example analyses and use of functions

### Reconstruction

For this paper, we will not run the analysis on a subject, but rather explore the output results for a subject included in the Freesurfer installation for reproducibility for the user. In particular, in the default Freesurfer subjects directory, there is a subject named “bert”. If we were to run all the analyses, we would use the recon_all code (described below):



recon_all(infile = "/path/to/T1.nii", subjid = "bert")
                    


We see the result of this output in the “bert” directory, which includes a series of sub-directories:



list.files(path  = file.path(fs_subj_dir(),  "bert"))

 [1] "bem"     "label"   "mri"     "scripts"  "src"    "stats"  "surf"
 [8] "tmp"     "touch"   "trash"
                    


We will explore the results in “mri”, which contain imaging data, “stats”, which containing statistics based on structures of the brain, and “surf”, which contain the surface and curvature output from the Freesurfer processing.

### MRI conversion: The mri convert function

The typical output format of brain volumes from Freesurfer is MGH/MGZ format, which is explained here: https://surfer.nmr.mgh.harvard.edu/fswiki/FsTutorial/MghFormat. As NIfTI formats are one of the most common formats and has been the common format for analysis in the **oro.nifti** and **neurobase** packages, it is useful to convert these files to a NIfTI format to read into R. The mri_convert Freesurfer function will be used for that conversion. Here we will use the T1-weighted image from the “bert” subject and convert it to NIfTI, and read it into R:



library(freesurfer)
bert_dir = file.path(fs_subj_dir(), "bert") # subject directory
t1_mgz = file.path(bert_dir, "mri", "T1.mgz") # mgz file
t1_nii_fname = tempfile(fileext = ".nii.gz") # temporary NIfTI file
freesurfer::mri_convert(t1_mgz, t1_nii_fname) # conversion
img = neurobase::readnii(t1_nii_fname) # read in outputs
                        
                    


As this is a commonly-used process, we have wrapped these two steps into the readmgz and readmgh functions, which combine the mri_convert and readnii functions. Here we show that these steps are equivalent to the readmgz function:



img_mgz = readmgz(t1_mgz)
identical(img, img_mgz)

[1] TRUE
                    


Now that we have the image in R, we can plot it using the standard plotting tools for nifti objects:



neurobase::ortho2(img, add.orient = FALSE, mask = img > 40)
                    


**Figure 1.**
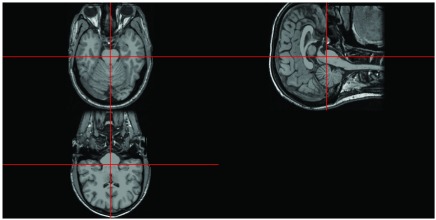
Plot of T1-weighted image from bert subject in Freesurfer.

The result is in Figure 1, which contains 3 slices of the head: axially, viewing the brain from the top of the head (top left), sagittally, viewing the brain from the right side (top right) and coronally, viewing the brain from the back of the head (bottom left).

Note, the image is not stored in the right/posterior/inferior (RPI) orientation which is assumed when displaying using the **neurobase**ortho2 function. We can use the rpi_orient function in **fslr** (version ≥ 2.4.0) ^15^ or fslswapdim to reorient the image to the assumed orientation.



L = fslr::rpi_orient(img)
reoriented_img = L[["img"]]
                    


We see that this function puts this image in the RPI orientation, which matches the assumed orientation for ortho2:



neurobase::ortho2(reoriented_img, mask = reoriented_img  >  40)
                    


**Figure 2.**
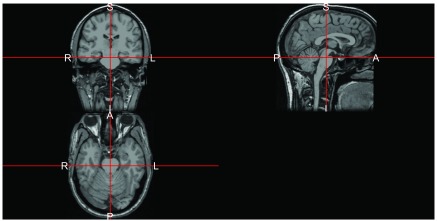
Plot of T1-weighted image from bert subject in Freesurfer after re-orientation to RPI orientation. Note, the letters denote the orientation of right/left (R/L), posterior/anterior (P/A), inferior/superior (I/S).

The result is in Figure 2, which changes the views in reference to which panel they are located and matches the orientation markers assumed by ortho2.

### Bias-field correction: The nu correct function

MRI images typically exhibit good contrast between soft tissue classes, but intensity inhomogeneities in the radio frequency field can cause differences in the ranges of tissue types at different spatial locations (e.g. top versus bottom of the brain). These inhomogeneities/non-uniformities can cause problems with algorithms based on histograms, quantiles, or raw intensities ^16^. Therefore, correction for image inhomogeneities is a crucial step in many analyses. The Freesurfer function nu_correct performs the non-uniformity correction by Sled *et al*. ^3^, and the **freesurfer** function of the same name will run the correction and return an image. The Freesurfer nu_correct function requires a MINC format ( http://www.bic.mni.mcgill.ca/ServicesSoftware/MINC). For this to work, you can convert the nifti object to a MINC file using nii2mnc:



mnc = nii2mnc(reoriented_img)
print(mnc)

[1] "/var/folders/1s/wrtqcpxn685_zk570bnx9_rr0000gr/T//RtmpHsAnD0/filec0c96f7f779e.mnc"
                    


We can pass this MINC file into the **freesurfer** nu_correct function, which will run the correction and then convert the output MNC to a NIfTI object.



nu_from_mnc = nu_correct(file = mnc)
class(nu_from_mnc)

[1] "nifti"
attr(,"package")
[1] "oro.nifti"
                    


**Figure 3.**
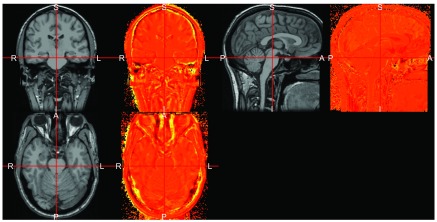
Inhomogeneity-corrected image output from Freesurfer nu_correct command and the estimated log bias-field.

We see that the results are indeed nifti objects. We can plot the estimated bias field (log-transformed for display purposes) side-by-side with the image to view which areas had been differentially corrected ( Figure 3).

In addition to the readmgz and readmgh functions above, we have a readmnc wrapper function for reading in MINC files, after conversion to NIfTI files. If you pass in a nifti object in directly into nu_correct, the function will automatically convert any NIfTI input files, and then run the correction (shown below). We can also pass in a mask of the brain (see next section) to run the correction only the areas of the brain.



nu_masked = nu_correct(file = reoriented_img, mask = mask)
class(nu_masked)

[1] "nifti"
attr(,"package")
[1] "oro.nifti"
                    


Overall, this correction is a way to make the intensities of the brain more homogeneous spatially. This method is different from that implemented in FSL ^13^ (and therefore **fslr**), so it provides an alternative method to the R user than currently available.

### Brain extraction: The mri watershed function

The mri_watershed function will segment the brain from the remainder of the image, such as extra-cranial tissues. Other imaging software in R have implemented the watershed algorithm, such as **EBImage** ^17^. These methods have not been directly adapted for MRI nor specifically for brain extraction. In **freesurfer**, we can pass in the nifti object and the output is a brain-extracted nifti object.



ss = mri_watershed(img)
ortho2(ss, mask = ss)
                    


**Figure 4.**
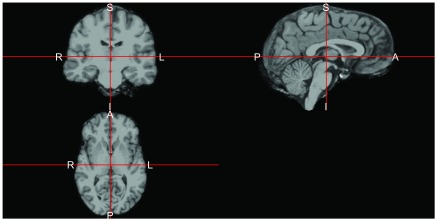
Brain-extracted image after using Freesurfer mri_watershed algorithm. We see that the areas outside of the brain have been removed from the image.

The result is in Figure 4, where we see areas of the skull, eyes, face, and other areas of the image are removed. We do see some areas remain that may be part of some of the membranes between the brain and the skull, but this looks like an adequate brain extraction for most analyses.

As the result is a nifti object, we can create a mask by standard logical operations for arrays. As MRI scans are typically positive-valued, the positive areas of the image are the “brain”:



mask = ss > 0
                    


We can then use this mask to perform operations on the image, such as subsetting.

### Segmentations of brain structures

Freesurfer is commonly used to segment cortical and subcortical structures of the brain. We can visualize images of these segmentations, which are located in the “mri” folder. We will choose the colors based on the Freesurfer look up table (LUT), which values can be explored at https://surfer.nmr.mgh.harvard.edu/fswiki/FsTutorial/AnatomicalROI/FreeSurferColorLUT. This look up table provides a label for each structure and the color associated with it:



head(freesurfer::fs_lut, 3)

  index                       label    R    G    B  A
1     0                     Unknown    0    0    0  0
2     1      Left-Cerebral-Exterior   70  130  180  0
3     2  Left-Cerebral-White-Matter  245  245  245  0
                    


This object is included in **freesurfer** and denotes the indices, labels, and color representation of the structure. We note that the alpha channel is set to 0 for all regions of interest, so we will not use it in the calculation of the colors from RGB space. This LUT allows visualizations produced in R to be consistent with those from Freesurfer.



seg_file = file = file.path(fs_subj_dir(), "bert", "mri", "aseg.mgz")
seg = readmgz(seg_file)
breaks = c(-1, fs_lut$index)
colors = rgb(fs_lut$R, fs_lut$G, fs_lut$B, alpha = 255/2, maxColorValue = 255)
ortho2(ss, seg, col.y = colors, ybreaks = breaks)
                    


**Figure 5.**
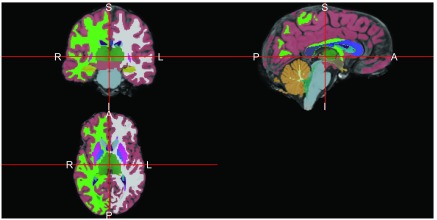
Overlay of segmentation from Freesurfer recon-all command.

Note above that the number of breaks must be one larger than the number of colors and the indices start at zero, so we add an additional element to the indices. The result in Figure 5 shows the image with colors overlaid.

### Reading in anatomical statistics for brain structures

We have explored the spatial results in the brain images, but not the quantitative information about the brain and sub-structures that are available from Freesurfer output. The “aseg.stats” in the “stats” folder for subject bert corresponds to measures and statistics from the anatomical segmentation. The read_aseg_stats function reads this corresponding file and creates a list of two different data.frames:



file = file.path(fs_subj_dir(), "bert", "stats", "aseg.stats")
out = read_aseg_stats(file)
names(out)

[1] "measures"  "structures"
                    


The measures element corresponds to global measurements of the brain (e.g. volume of the brain) as well as measures of gross anatomical structures (e.g. gray matter).



head(out$measures[, c("meaning", "value", "units")], n = 3)

                                                 meaning	  value
1                              brain segmentation volume 1193318.000000
2           brain segmentation volume without ventricles 1174082.000000
3 brain segmentation volume without ventricles from surf 1173867.217735 
  units
1  mm^3
2  mm^3
3  mm^3
                    


In some imaging analyses, comparing at these large measures of brain volume over time or across groups are of interest. Alternatively, the structures element corresponds to a set of measures and statistics for a set of fixed anatomical structures.



head(out$structures, n = 3)

 Index SegId NVoxels Volume_mm3                  StructName normMean
1    1     4    6563     6562.6       Left-Lateral-Ventricle 36.0959
2    2     5     228      228.3            Left-Inf-Lat-Vent 54.8842
3    3     7   15708    15708.2 Left-Cerebellum-White-Matter 92.7562
 normStdDev normMin normMax normRange
1   12.2771      16      91        75
2   10.7839      22      87        65
3    5.5123      40     107        67
                    


Similarly with global measures, these structure-specific measures are used in analysis. For example, measuring differences in hippocampus volumes across patients with Alzheimer’s disease and those without. Moreover, a large deviation in volume, globally or locally, for a specific subject may indicate atrophy of a structure or an indication of a segmentation error.

### Converting surfaces using mris convert

**Figure 6.**
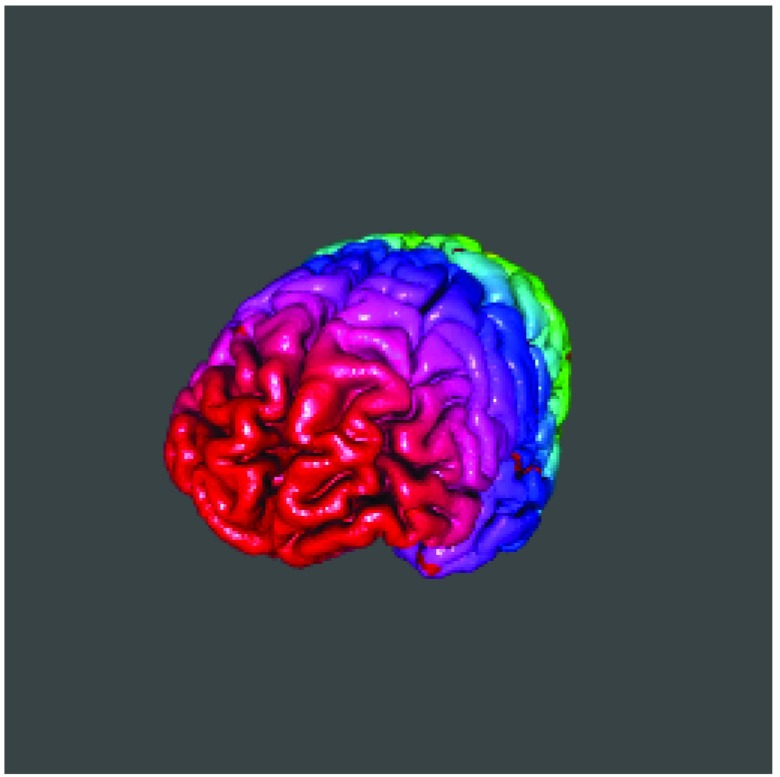
Pial surface from bert subject in Freesurfer rendered using rgl.



Freesurfer includes segmentations of different surfaces of the brain alongside the volumetric segmentations above. As the mri_convert function provides a tool to convert image volumes to a series of output formats, the mris_convert (note the “s”) allows users to convert between image surface formats. These surfaces usually store sets of vertices and faces to be plotted in 3 dimensions. **freesurfer** has implemented mris_convert (with a function of the same name) as well as functions to convert surfaces from Freesurfer to a set of triangles in R, such as surface_to_triangles. We will read in the left and right side of the pial surface of the brain and display the surface using rgl ^18^ ( Figure 6).

right_file = file.path(fs_subj_dir(),
                   "bert", "surf", "rh.pial")
right_triangles = surface_to_triangles(infile = right_file)
left_file = file.path(fs_subj_dir(),
                   "bert", "surf", "lh.pial")
left_triangles = surface_to_triangles(infile = left_file)
rgl::rgl.open()
rgl::rgl.triangles(right_triangles,
                   color = rainbow(nrow(right_triangles)))
rgl::rgl.triangles(left_triangles,
                   color = rainbow(nrow(left_triangles)))
                    


Thus, we can read in the output images, surfaces, and the tables of output metrics from Freesurfer.

### Additional features

For the initial release, we did not implement a method to read the annotation files and other surface-based files that Freesurfer uses. Reading in these files are planned for a future release and may work with the functions described above. Freesurfer can also analyze diffusion tensor imaging data and some of the functions have been adapted for **freesurfer** but have not been thoroughly tested.

## Conclusion

The neuroimaging community has developed a large collection of tools for image processing and analysis. These tools have additional functionality that is not present in R, such as the surface-based registration and processing Freesurfer provides. We have provided a similar incorporation of tools from FSL to R in the **fslr** package and have repeated the effort for Freesurfer with the **freesurfer** package to bridge this gap and provide R users functions from Freesurfer.

There has been an increasing popularity of similar interfacing of tools within the Python community such as Nipype ^19^. As many users of R may not have experience with Python or bash scripting, we believe **freesurfer** provides a lower threshold for use in the R community.

Lowering this threshold is important because it allows more R users to control all aspects of image analysis from raw image processing to final statistical analysis. Interfacing R with existing, powerful software provides R users more functionality and a additional support community, which would not be available if the functions were rewritten in R. Although having an external software dependency may be disadvantage to R users, the software used benefits from the years of previous testing. Most importantly, as **freesurfer** is based on the R framework, all the benefits of using R are available, such as dynamic documents, Shiny applications, customized figures, and state-of-the-art statistical methods. This added functionality affords the neuroimaging and R communities the ability to have analysis in one framework while borrowing the strengths of both.

## Experimental features

### Label files

Although we have not thoroughly tested reading in a label file from Freesurfer, we have provided the read_fs_label function. Here we will read a label file for the left hemisphere cortex:



file = file.path(fs_subj_dir(), "bert", "label", "lh.cortex.label")
out = read_fs_label(file)
head(out)

 vertex_num r_coord  a_coord  s_coord        value
1         0 -12.882 -102.449   -9.782 0.0000000000
2         1 -13.331 -102.518   -9.829 0.0000000000
3         2 -13.637 -102.514  -10.077 0.0000000000
4         3 -13.031 -102.596  -10.024 0.0000000000
5         4 -13.331 -102.510  -10.254 0.0000000000
6         5 -13.610 -102.483  -10.295 0.0000000000
                    


## Software availability

**freesurfer** is available at: https://cran.r-project.org/package=freesurfer

Source code is available at: https://github.com/muschellij2/freesurfer

Archived source code as at time of publication: http://doi.org/10.5281/zenodo.1213308 ^20^

Software license: GPL-2

All necessary code to generate this report is located at: https://github.com/muschellij2/fs_paper.
