# Supplementary material for: freesurfer: Connecting the Freesurfer software with R
Source: F1000Res. 2018 May 16;7:599. [Version 1] doi: 10.12688/f1000research.14361.1 (PMC6051196; doi:10.12688/f1000research.14361.1)
Supplement: Peer review history

# Peer review: Referee response for version 1

**DOI:** 10.5256/f1000research.15624.r35180

**Published:** 2018-07-16

**Authors:** Pierre Lafaye de Micheaux

**Affiliations:** 1 School of Mathematics and Statistics, University of New South Wales, Sydney, Australia

This article describes a very interesting R package that enables R users to have access, directly from within R, to several functions from the great neuroimaging open source software Freesurfer. The paper is well written, and their package useful.

 I only have minor comments to improve the readability of the paper.

 First, I would suggest to use brackets () to indicate freesurfer R functions and avoid confusion with Freesurfer command line functions. For example recon_all() for freesurfer and recon_all fro Freesurfer.

 Next, I tried to replicate all the results presented in the paper. My OS is Linux Debian 9.3 Stretch on a DELL laptop.

 First, I tried to install Freesurfer, following the link given in the paper. Two commands I had to use to make things work are:

 sudo apt-get install tcsh

 sudo ln -s /usr/lib/x86_64-linux-gnu/libpng16.so /usr/lib/x86_64-linux-gnu/libpng12.so.0

 Then I wanted to install FSL. I think the authors of the current paper should give a URL (e.g., https://fsl.fmrib.ox.ac.uk/fsldownloads_registration) to indicate where to download FSL. Unfortunately, FSL binaries are not available (yet) for Debian 9. I tried to install from the sources with no success. I then went back to the same URL and chose Debian 8, to download the fslinstaller.py file, then run the following command:

 python fslinstaller.py

 This finally worked as expected. The authors of the current paper might want to indicate that these installation can take quite some time.

 I then launched an R session directly from the console, and typed the instructions given in the paper to check if they all work fine.

 I would suggest to give the following instructions before the user have to type all the instructions given in the paper. It was obvious for me, but might not be so for less experienced R users:

 install.packages("fslr")

 install.packages("freesurfer")

 install.packages("rgl")

 install.packages("neurobase")

 I suggest that close to this sentence " If we were to run all the analyses, we would use the recon_all code (described below):", the authors indicate that this is not done since it would take more than 20 hours (if this is indeed the case). 

 I goth this first error:

 > L = fslr::rpi_orient(img)

 Error in get.fsl() : Can't find FSL

 In addition: Warning message:

 In get.fsloutput() : Can't find FSLOUTPUTTYPE, setting to NIFTI_GZ

 I then typed:

 > fslr::have.fsl()

 [1] FALSE

 I solved this error with:

 > options(fsl.path="/home/lafaye/fslbuild/fsl")

 > fslr::have.fsl()

 [1] TRUE

 Of course, this is because, due to hard-disk space constraints, I installed FSL (and also Freesurfer) in my home directory instead of the standard location. Maybe this could be mentioned.

 When I typed this instruction:

 > nu_from_mnc = nu_correct(file = mnc)

 it worked but I got this warning:

 Malformed NIfTI - not reading NIfTI extension, use at own risk!

 When I typed this instruction:

 > nu_masked = nu_correct(file = reoriented_img, mask = mask)

 I got this error:

 Error in nu_correct(file = reoriented_img, mask = mask) : 

   object 'mask' not found

 Indeed, the object 'mask' is defined later on. I suggest to create this 'mask' object first, or otherwise to type the above instruction later.

 This instruction also failed:

 > ortho2(ss, mask = ss)

 Error: could not find function "ortho2"

 One should use (if the package neurobase is not loaded):

 > neurobase::ortho2(ss, mask = ss)

 > mask = ss >0

 I then retried the previous instruction now we have the mask:

 > nu_masked = nu_correct(file = reoriented_img, mask = mask)

 but I got this error:

 Direction cosines of /tmp/RtmpxVKjYw/file29ad28e1ded7.mnc and /var/tmp/nu_correct_11320/file29ad516b90fd.mnc do not match

 Failed to shrink mask volume.

 nu_correct: crashed while running nu_estimate_np_and_em (termination status=65280)

 mnc2nii -float "/tmp/RtmpxVKjYw/file29ada6796e2.mnc"   "/tmp/RtmpxVKjYw/file29ad2a6e70b2.nii";

 Can't find input file '/tmp/RtmpxVKjYw/file29ada6796e2.mnc'

 Error in mnc2nii(tmpfile, outfile = outfile) : 

   mnc2nii did not produce outfile specified

 Not sure what to do here ...

 Apart form that, everything was fine!

I have read this submission. I believe that I have an appropriate level of expertise to confirm that it is of an acceptable scientific standard.

# Peer review: Referee response for version 1

**DOI:** 10.5256/f1000research.15624.r35181

**Published:** 2018-07-06

**Authors:** Seth Lirette

**Affiliations:** 1 Department of Data Science, University of Mississippi Medical Center, Jackson, MS, USA

Muschelli, Sweeney, and Crainiceanu have provided researchers (and particularly statisticians) with a useful new tool for analyzing structural neuroimaging data. They cite a number of packages for analyzing and manipulating imaging data, but lack the functionality of Freesurfer. They well document the processes for reading and converting file formats to obtain usable R objects. The examples for plotting, bias-field correction, surface mapping, and brain extraction are clear, concise, and provide readers with enough information to recreate these with their own data. A very minor addition I would like to see would be an example of how to extract the volumetric and segmented volumetric data from a sample of subjects (or from multiple studies on the same subject). These could then be stored in other R objects (vectors, data frames, etc.) to be further analyzed using statistical methodologies. Overall, this is a very nice tool, and I personally am excited to use **freesurfer **very soon.

I have read this submission. I believe that I have an appropriate level of expertise to confirm that it is of an acceptable scientific standard.
